# Small proteins in cyanobacteria provide a paradigm for the functional analysis of the bacterial micro-proteome

**DOI:** 10.1186/s12866-016-0896-z

**Published:** 2016-11-28

**Authors:** Desiree Baumgartner, Matthias Kopf, Stephan Klähn, Claudia Steglich, Wolfgang R. Hess

**Affiliations:** 1 University of Freiburg, Faculty of Biology, Genetics and Experimental Bioinformatics, Schänzlestr. 1, D-79104 Freiburg, Germany; 2 Present Address: Molecular Health GmbH, Kurfürsten-Anlage 21, 69115 Heidelberg, Germany

**Keywords:** Cyanobacteria, Nitrogen deprivation, Photosynthesis, *Synechocystis*, Small proteins

## Abstract

**Background:**

Despite their versatile functions in multimeric protein complexes, in the modification of enzymatic activities, intercellular communication or regulatory processes, proteins shorter than 80 amino acids (μ-proteins) are a systematically underestimated class of gene products in bacteria. Photosynthetic cyanobacteria provide a paradigm for small protein functions due to extensive work on the photosynthetic apparatus that led to the functional characterization of 19 small proteins of less than 50 amino acids. In analogy, previously unstudied small ORFs with similar degrees of conservation might encode small proteins of high relevance also in other functional contexts.

**Results:**

Here we used comparative transcriptomic information available for two model cyanobacteria, *Synechocystis* sp. PCC 6803 and *Synechocystis* sp. PCC 6714 for the prediction of small ORFs. We found 293 transcriptional units containing candidate small ORFs ≤80 codons in *Synechocystis* sp. PCC 6803, also including the known mRNAs encoding small proteins of the photosynthetic apparatus. From these transcriptional units, 146 are shared between the two strains, 42 are shared with the higher plant *Arabidopsis thaliana* and 25 with *E. coli*. To verify the existence of the respective μ-proteins in vivo, we selected five genes as examples to which a FLAG tag sequence was added and re-introduced them into *Synechocystis* sp. PCC 6803. These were the previously annotated gene *ssr1169*, two newly defined genes *norf1* and *norf4*, as well as *nsiR6* **(n**itrogen **s**tress-**i**nduced **R**NA **6) ** and *hliR1*(**h**igh **l**ight-**i**nducible **R**NA **1**) , which originally were considered non-coding. Upon activation of expression via the Cu^2+.^responsive *petE* promoter or from the native promoters, all five proteins were detected in Western blot experiments.

**Conclusions:**

The distribution and conservation of these five genes as well as their regulation of expression and the physico-chemical properties of the encoded proteins underline the likely great bandwidth of small protein functions in bacteria and makes them attractive candidates for functional studies.

## Background

Proteins with less than 80 amino acids in prokaryotes or 100 amino acids in eukaryotes are defined as short proteins (μ-proteins). During standard genome annotation these short protein-coding genes are frequently neglected and proteomics-based analyses fail to detect this class of peptides routinely. As a result, μ-protein-coding genes are a systematically underestimated class of gene products.

In strong contrast is the finding that small ORFs constitute the most frequent essential genomic component in bacteria, even more than conventional ORFs [1]. Indeed, the functional characterization of selected examples of μ-proteins has revealed their critical involvement in processes such as quorum sensing or interspecies communication [2], regulatory functions [3–6] and in the formation of multi-subunit protein complexes. An increasing number of μ-proteins is being discovered also in eukaryotes [7–10], and archaea [11], indicating their ubiquity in all three domains of life. Nevertheless, the likely diverse functions of short proteins are largely unknown, even for simple unicellular bacteria.

Photosynthetic cyanobacteria provide a paradigm for small protein functions due to extensive work on the photosynthetic apparatus that led to the functional characterization of 19 μ-proteins of less than 50 amino acids, that play a role in photosystem II (genes *psbM*, *psbT* (*ycf8*), *psbI*, *psbL*, *psbJ*, *psbY*, *psbX*, *psb30* (*ycf12*), *psbN*, *psbF*, *psbK* [12, 13]), in photosystem I (*psaM*, *psaJ*, *psaI* [14]), photosynthetic electron transport (Cyt*b*_6_ *f* complex; *petL*, *petN*, *petM*, *petG* [15–17]), or have accessory functions (*hliC* (*scpB*) [18]). The shortest annotated protein conserved in cyanobacteria is with 29 amino acids the cytochrome *b*_6_ *f* complex subunit VIII, encoded by *petN* [19].

Several cyanobacterial model species have been studied by transcriptomics [20–28] and proteomics [29–31] approaches but there are no reports specifically targeting μ-proteins. Based on extensive comparative transcriptome and genome information we used the model cyanobacterium *Synechocystis* sp. PCC 6803 (*Synechocystis* 6803) and the closely related strain *Synechocystis* sp. PCC 6714 (*Synechocystis* 6714) [20–22, 32] for the prediction of possible μ-ORFs. We found 293 transcriptional units (TU) containing candidate small ORFs ≤80 codons in *Synechocystis* 6803, including all known mRNAs encoding small proteins of the photosynthetic apparatus.

We chose 5 examples from *Synechocystis* 6803 for experimental analysis. These were *norf1* and *norf4* (for novel orf 1 and 4, [22]), *nsiR6* and *hliR1* (for nitrogen stress-induced RNA 6 and high light inducible RNA 1), the latter two transcripts originally considered non-coding [33] as well as the short gene *ssr1169*, which was predicted as protein-coding in the current version of the genome sequence [NCBI reference NC_000911]. All five proteins could be detected after FLAG tagging in vivo. Their modes of regulation, conservation and physico-chemical properties make these five μ-proteins interesting candidates for functional studies.

## Methods

### Strains and growth conditions

*Synechocystis* 6803, substrain “PCC-M” [34], served as WT and was grown in Cu^2+^-free, TES-buffered (20 mM, pH 8.0) liquid BG11 medium [35] with gentle agitation or on agar-solidified (0.9% [w/v] Kobe I agar, Roth, Germany) BG11 supplemented with 0.3% (w/v) sodium thiosulfate at 30 °C under continuous illumination with white light of ~40 μmol photons m^−2^ s^−1^. To induce expression of FLAG - tagged μ-proteins from the Cu^2+^-responsive *petE* promoter [36] 2 μM CuSO_4_ was added to exponentially growing cells. Different environmental conditions were applied for induction of gene expression under control of native promoters: (i) high light, 300 μmol photons m^−2^ s^−1^; (ii) dark, flasks wrapped with aluminium foil; (iii) nitrogen deficiency, cells were pelleted by centrifugation, washed once and resuspended in NO_3_^−^-free BG11. Samples for protein extraction were taken just before and 6 h (Norf1, HliR1) or 24 h (NsiR6, Norf4) after induction of gene expression. Ssr1169 was expected to be most expressed in exponential growth phase, hence samples were taken from exponentially growing cells at two consecutive days. *Synechocystis* 6803 strain pUR-PpetJ-3xFlag-sfGFP [37] was used as positive control for the detection of FLAG-tagged proteins by Western blots. *E. coli* strains TOP10F’ and J53/RP4 were used for generating *Synechocystis* 6803 mutant strains by conjugation. In liquid BG11 medium 5 μg ml^−1^ gentamicin or 50 μg ml^−1^ kanamycin and 5 μg ml^−1^ gentamicin were used to maintain recombinant strains (see below).

For examination of gene expression by Northern blot analysis, exponentially growing WT cells were transferred to the different environmental conditions described above. Cultivation under high light was followed by a shift back to standard light conditions (40 μmol photons m^−2^ s^−1^)). Cultures grown in the dark as well as nitrogen deprived cultures were additionally aerated with ambient air through a glass tube and a sterile filter for constant and fast growth.

### Computational methods

Small ORFs and their orthologs were identified and annotated in *Synechocystis* 6803 and 6714 in three steps.

1. BlastN searches returning hits with E values ≤1e^−2^ were performed against the NCBI nt database [38] for all intergenic regions covered by TUs [20, 21]. From the blast results, multiple alignments were created with ClustalW [39] and analyzed for their coding potential with RNAcode [40]. The significant (*p* ≤0.05) small ORF candidates were manually curated.
2. To annotate candidate small ORFs, blastP queries with E values ≤1e^−2^ were done against the NCBI nr database [38].
3. Orthologs of existing and newly detected small ORFs were identified in *Synechocystis* 6803 and 6714 via a reciprocal best hit approach using blastP with a minimum E value ≤1e^−2^ and allowing a difference in length of ≤20% and a maximum length of 80 amino acids in both strains.

Genes of small ORFs that were covered by a predicted TU were considered to be expressed. Transmembrane helices were predicted with TMHMM Server v. 2.0 [41].

### Generation of mutant strains

Gene constructs for ectopic expression of FLAG - tagged Norf1 under control of the *petE* promoter or the native promoter were generated via gene synthesis (Eurofins). The constructs consisted of the upstream sequence of *petE* (P*petE* = −273 to +100 referring to the first transcribed nucleotide as +1) or the upstream sequence of *norf1* (P*norf1* = −328 to +143), the *norf1* coding sequence omitting the stop codon (+144 to +287, corresponding to genome positions 298829 to 298972), a 3xFLAG coding tag (sequence: ATGGATTATAAAGATCATGATGGCGATTATAAAGATCATGATATTGATTATAAAGATGATGATGATAAA) followed by a stop codon (TAG), the *norf1* 3′UTR (+291 to +425) and the bacteriophage lambda oop terminator. The obtained P*petE*::*norf1*::3xFLAG::T*oop* and P*norf1*::*norf1*::3xFLAG::T*oop* constructs were digested with *Xho*I and *Hin*dIII and introduced into self-replicating vector pVZ322 [42]. The resulting plasmids were transferred into *Synechocystis* 6803 WT via triparental mating with *E. coli* J53/RP4 and TOP10F’ [43]. These two recombinant strains were selected on BG11 agar containing 10 μg ml^−1^ gentamicin.

To establish ectopic expression of FLAG - tagged NsiR6, HliR1, Ssr1169 and Norf4, the respective genomic sequences (*nsiR6* 729671 to 729868, *hliR1* 1606868 to1606978, *ssr1169* 3084421 to 3084582, *norf4* 2425146 to 2425238) were amplified using the primer pairs nsiR6_fw/nsiR6_rev, PpetE::hliR1_fw/3xFlag_hliR1_rev, PpetE::ssr1169_fw/3xFlag_ssr1169_rev and PpetE::Norf4_fw/3xFlag_Norf4_rev. All oligonucleotides used in this study are listed in Table 1. The *petE* promoter was amplified separately for each construct to generate overlaps with the particular μ-ORFs using the primer pUC19-XbaI_PpetE_fw in different combinations with nsiR6::PpetE_rev, hliR1::PpetE_rev, ssr1169::PpetE_rev or Norf4::PpetE_rev. The 3′ segments consisting of the sequence encoding the 3xFLAG tag (+ stop codon TAG), the 3′UTR of the *norf1* mRNA and the oop terminator were amplified from the plasmid obtained via gene synthesis described above using the primer 3xFlag_PstI-pUC19_rev in combination with nsiR6_3xFlag_fw, hliR1_3xFlag_fw, ssr1169_3xFlag_fw or Norf4_3xFlag_fw, respectively. Fragments belonging together were combined by Gibson Assembly® Master Mix (New England Biolabs) according to the manufacturer’s instructions utilizing *Xba*I and *Pst*I digested pUC19 as vector backbone. For expression of the small proteins under control of their native promoters the obtained plasmids served as templates for amplifying corresponding coding sequences associated with the 3′ segment described above using the primer 3xFlag_PstI-pUC19_rev in combination with CDSnsiR6::PnsiR6_fw, CDShliR1::PhliR1_fw, CDSnorf4::Pnorf4 or CDSssr1169::Pssr1169_fw. Upstream sequences of *nsiR6*, *hliR1*, *norf4* and *ssr1169* considered as promoter sequences (P*nsiR6* = 729258 to 729670, P*hliR1* = 1606503 to 1606867, P*norf4* = 2424768 to 2425145, P*ssr1169* 3084025 to 3084420) were amplified from *Synechocystis* 6803 genomic DNA with the primer pairs pUC19::PnsiR6_fw/PnsiR6::CDSnsiR6_rev, pUC19::PhliR1_fw/PhliR1::CDShliR1_rev, pUC19::Pnorf4_fw/Pnorf4::CDSnorf4_rev or pUC19::Pssr1169_fw/Pssr1169::CDSssr1169_rev. Related fragments were combined by Gibson Assembly® Master Mix as described above. All resulting cassettes were released by restriction, introduced into pVZ322 [42] and transferred into *Synechocystis* 6803 WT via triparental mating. Additionally, the empty vector pVZ322 was introduced into the wild type to create a control strain. The recombinant strains were selected on BG11 agar containing 10 μg ml^−1^ gentamicin and 50 μg ml^−1^ kanamycin.

**Table 1:** List of oligonucleotides

Name of Oligonucleotide	Sequence (in 5′ – 3′ direction)	Application
Probe_norf1_fw	GTAATACGACTCACTATAGGGAGACCATCGACTATTCTTCAGTACTGTTTAC	Amplification of probe template *norf1* (168 nt) for in vitro-transcription (T7 promoter is underlined)
Probe_norf1_rev	TTGAGATGCTACAGGACCTTATGC	
Pnorf1_fw	taccggtGCCTAGGGGATACCTCTCCCC	Amplification of putative *norf1* promoter for ligation into pILA reporter plasmid
Pnorf1_rev	tggccggcCTCCGTCCCAATGGGGGAAAC	
nsiR6_probe_fw	GTAATACGACTCACTATAGGGAGATTACCGATCGCCGCTTCATC	Amplification of probe template *nsiR6* (166 nt) for in vitro-transcription (T7 promoter is underlined)
nsiR6_probe_rev	TGTGTGGCGTCACCATTGAAAATG	
hliR1_probe_fw	GTAATACGACTCACTATAGGGAGACTCGGGAAGATTAAGACTGGTTTTG	Amplification of probe template *hliR1* (135 nt) for in vitro-transcription (T7 promoter is underlined)
hliR1_probe_rev	ATGTCTAATTTGATTGCTGTTGCTTTC	
norf4_probe_fw	GTAATACGACTCACTATAGGGAGACCCCCTTTAGCAAAACTACCCATC	Amplification of probe template *norf4* (116 nt) for in vitro-transcription (T7 promoter is underlined)
norf4_probe_rev	ATGACCGCCGATCAACTGTTG	
nsiR6_fw	gccaagaagtATGAGTGTTTTCCCCGCAGA	Amplification of *nsiR6* generating overlaps with P*petE* and 3xFLAG::3′UTR *norf1*::T*oop*
nsiR6_rev	tataatccatGTCGTAATAATCCCGGCTGG	
PpetE::hliR1_fw	gccaagaagtATGTCTAATTTGATTGCTGTTG	Amplification of *hliR1* generating overlaps with P*petE* and 3xFLAG::3′UTR *norf1*::T*oop*
3xFlag_hliR1_rev	tataatccatCTCGGGAAGATTAAGACTGG	
PpetE::ssr1169_fw	gccaagaagtATGGATATTGTTAAGATCATTTGTGCGATTC	Amplification of *ssr1169* generating overlaps with P*petE* and 3xFLAG::3′UTR n*orf1*::T*oop*
3xFlag_ssr1169_rev	tataatccatACGTTCCCTGGCAATGACCC	
PpetE::Norf4_fw	gccaagaagtATGACCGCCGATCAACTGTT	Amplification of *n* *orf4* generating overlaps with P*petE* and 3xFLAG::3′UTR *n* *orf1*::T*oop*
3xFlag_Norf4_rev	tataatccatACCCCCTTTAGCAAAACTAC	
pUC19-XbaI_PpetE_fw	gctcggtacccggggatcctctagaCTGGGCCTACTGGGCTATTC	Amplification of P*petE* introducing *Xba*I site (underlined) + creating overlaps with pUC19 and *nsiR1*, *hliR* *1*, *ssr1169* or *n* *orf4*
nsiR6::PpetE_rev	aaacactcatACTTCTTGGCGATTGTATCTATAGG	
hliR1::PpetE_rev	aattagacatACTTCTTGGCGATTGTATCTATAGG	
ssr1169::PpetE_rev	caatatccatACTTCTTGGCGATTGTATCTATAGG	
Norf4::PpetE_rev	gatcggcggtcatACTTCTTGGCGATTGTATCTATAGG	
3xFlag_PstI-pUC19_rev	gccaagcttgcatgcctgcagAATAAAAAACGCCCGGCGGC	Amplification of 3xFLAG::3′UTR *norf1*::T*oop* introducing *Pst*I site (underlined) + creating overlaps with pUC19 and *nsiR1*, *hlirR*, *ssr1169* or *norf4* or amplification of particular CDS associated with 3xFLAG__3′UTR *norf1*::T*oop* introducing *Pst*I site (underlined) + creating overlaps with pUC19 and respective promoter sequence
nsiR6_3xFlag_fw	ttattacgacATGGATTATAAAGATCATGATGGCGATTATAAAG	
hliR1_3xFlag_fw	tcttcccgagATGGATTATAAAGATCATGATGGCGATTATAAAG	
ssr1169_3xFlag_fw	cagggaacgtATGGATTATAAAGATCATGATGGCGATTATAAAG	
Norf4_3xFlag_fw	taaagggggtATGGATTATAAAGATCATGATGGCGATTATAAAG	
CDSnsiR6::PnsiR6_fw	ataaatactcATGAGTGTTTTCCCCGCAGAAAC	
CDShliR1::PhliR1_fw	aaattaactaaATGTCTAATTTGATTGCTGTTGCTTTCTG	
CDSNorf4::PNorf4_fw	aatttttaccATGACCGCCGATCAACTGTTG	
CDSssr1169::Pssr1169_fw	gagtgaactaATGGATATTGTTAAGATCATTTGTGCGATTC	
pUC19::PnsiR6_fw	gctcggtacccggggatcctctagaATCGCCGTATTACACCTCTG	Amplification of P*nsiR6* introducing *Xba*I site (underlined) + generating overlaps with pUC19 and *nsiR6*.
PnsiR6::CDSnsiR6_rev	aaacactcatGAGTATTTATTCCTAGTGAATGAATTAGAAG	
pUC19::PhliR1_fw	gctcggtacccggggatcctctagaGGAGTTTACAGCGAGATTTG	Amplification of P*hliR1* introducing *Xba*I site (underlined) + generating overlaps with pUC19 and *hliR1*.
PhliR::CDShliR1_rev	aattagacatTTAGTTAATTTTTGTAACGGGAG	
pUC19::PNorf4_fw	gctcggtacccggggatcctctagaAGGTGATGATTATGAGCCGTC	Amplification of P*norf4* introducing *Xba*I site (underlined) + generating overlaps with pUC19 and *norf4*.
PNorf4::CDSNorf4_rev	gatcggcggtcatGGTAAAAATTCCACTAATTCAAAAAAC	
pUC19::Pssr1169_fw	gctcggtacccggggatcc*tctaga*CGAGTAGCCAGCCAAAGCAG	Amplification of P*ssr1169* introducing *Xba*I site (underlined) + generating overlaps with pUC19 and *ssr1169*.
Pssr1169::CDSssr1169_rev	caatatccatTAGTTCACTCCAATATGTCGGGATAATTAG	

### RNA extraction and analysis

*Synechocystis* 6803 cells were harvested by vacuum filtration on hydrophilic polyethersulfone filters (Pall Supor®-800, 0.8 μm), immediately immersed in 1 ml PGTX [44] and frozen in liquid nitrogen. RNA extraction was performed by 15 min incubation at 65 °C followed by chloroform washing and isopropanol precipitation as previously described [45]. Northern hybridization with ^32^P-labelled, single-stranded transcript probes was carried out as described [46]. Oligonucleotide sequences for PCR amplification of probe templates used for in vitro transcription are listed in Table 1.

### Protein purification and immunodetection

Cells for protein extraction were collected by centrifugation (4000 × g, 10 min, 4 °C), resuspended in PBS buffer (137 mM sodium chloride, 2.7 mM potassium chloride, 10 mM disodium phosphate, 1.8 mM potassium dihydrogen phosphate, pH 7.4) in the presence of protease inhibitor cocktail (cOmplete, Roche) and immediately frozen in liquid nitrogen. Cells were mechanically disrupted by using glass beads (diameter 0.1–0.25 mm) and a Precellys® 24 homogenizer (Bertin Technologies) at 6000 rpm and 4 °C applying six cycles of 3 × 10 s homogenization. Glass beads were removed by centrifugation (1000 × g, 1 min, 4 °C). To solubilize membrane proteins, samples were heated for 30 min at 50 °C with 2% SDS (w/v) followed by determination of the protein concentration using Direct Detect Spectrometer (Merck Millipore).

Proteins were separated by SDS-PAGE on 15% (w/v) polyacrylamide gels and stained with GelCode® Blue Stain Reagent (Thermo Scientific). PageRuler™ Prestained Protein Ladder (10–170 kDa, Fermentas) was used as molecular weight marker.

For immunoblot analysis, separated proteins were transferred to nitrocellulose membranes (Hybond™-ECL, GE Healthcare). Membranes were blocked over night at 4 °C with 5% low fat milk powder in TBS-T and subsequently probed with monoclonal ANTI-FLAG® M2-Peroxidase (HRP) antibody raised in mouse (Sigma-Aldrich) in TBS-T for 1 h at room temperature in the dark. All washing steps were performed with gentle agitation in TBS-T (20 mM Tris pH 7.6, 150 mM NaCl, 0.1% (v/v) Tween-20) at room temperature. Signals were detected with ECL™ start Western blotting detection reagent (GE Healthcare) on a chemiluminescence imager system (Fusion SL, Vilber Lourmat) and subsequently visualized using FUSION-CAP (Vilber Lourmat) and Quantity One software (BIO-RAD).

### Reporter gene assays

To measure promoter activity as a function of bioluminescence the putative *norf1* promoter sequence and its 5′UTR (−328 to +137, TSS at +1) was fused to promoterless *luxAB* reporter genes by PCR, followed by cloning into the promoter probe vector pILA as described [47]. The resulting pILA derivative was used for transformation of a *Synechocystis* 6803 strain expressing the *luxCDE* genes encoding enzymes for the synthesis of decanal, the luciferase substrate, under control of the strong promoter of the ncRNA Yfr2a [48].

Cells were grown in the presence of 10 mM glucose to provide energy for the luciferase reaction also in darkness. Bioluminescence was measured in vivo at different time points after inducing dark conditions as described [47].

## Results

### Comparative transcriptomics for the identification of μ-proteins in *Synechocystis*

The extensive comparative transcriptome and genome information for the model cyanobacterium *Synechocystis* 6803 [21, 22] and the closely related strain *Synechocystis* 6714 [20, 32] was utilized for the prediction of possible μ-ORFs. In our previous studies [20, 21] transcriptional units (TUs) had been defined, combining information on the transcriptional start sites, the lengths of transcribed UTRs, operons, coding and non-coding regions.

**Fig. 1:**
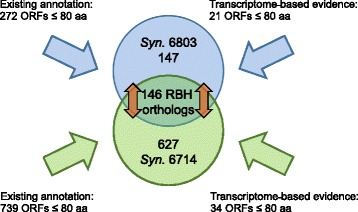
Scheme of computational prediction of small ORFs in cyanobacteria. Small ORFs were detected based on the transcriptome information for expressed intergenic regions [20–22] and their coding potential evaluated with RNAcode [40]. This information was merged with the pre-existing annotation [32]. Orthologs between the two small ORF populations were detected when they were identified as reciprocal best hits (RBH) by blastP with e ≤ 1e^−2^

Here we judged all possible non-coding transcripts by the program RNAcode [40] for their protein-coding potential. RNAcode detects protein-coding regions in any given sequence on the basis of multiple sequence alignments and the evolutionary signatures that are associated with a coding sequence [40]. After combination with the pre-existing annotation, this analysis led to the prediction of 293 potential small proteins with a maximum of 80 amino acids in *Synechocystis* 6803 and possibly 773 in *Synechocystis* 6714 (Fig. 1).

The resulting sets of candidate μ-proteins were compared against the predicted proteome of the respective other *Synechocystis* strain, against *E. coli* and the higher plant *Arabidopsis thaliana* as reference organisms for proteins possibly conserved among bacteria or among photosynthetic organisms. This procedure led to the identification of 146 μ-proteins shared between the two *Synechocystis* strains, as well as 42 and 29 μ-proteins which are shared between *Synechocystis* 6803 and *A. thaliana* or *E. coli*, respectively. Interestingly, we found the 42 proteins shared with higher plants to be identical in both *Synechocystis* strains. In contrast to observations in other bacteria, a relatively high number of the predicted proteins in the smallest fraction (≤50) had assigned functions (e.g., in photosynthesis) and a matching protein in the higher plant *Arabidopsis thaliana* or in *E. coli* (Table 2).

**Table 2:** Predicted and previously annotated proteins ≤50 amino acids in *Synechocystis* 6803

Start	End	S	L	Locus_tag	Gene	Product	6714	A. th.	E. coli
608828	608748	-	26	Chr_ORF_4	NA	Hypothetical protein	N	N	N
1840846	1840926	+	26	Chr_ORF_10	NA	Hypothetical protein	Y	N	N
160004	160093	-	29	sml0004	*petN*	Cytochrome *b*_6_ *f* complex subunit VIII	Y	Y	N
586617	586525	-	30	Chr_ORF_3	NA	Hypothetical protein	Y	N	N
2414584	2414679	+	31	smr0001	**psbT, ycf8**	Photosystem II PsbT protein	Y	Y	N
467201	467296	+	31	smr0005	**psaM**	Photosystem I subunit XII	Y	N	N
**1148230**	**1148325**	**-**	**31**	**Norf4**	**norf4**	**Norf4**	**Y**	**N**	**N**
1643502	1643600	-	32	ssl3803	*petL*	Cytochrome *b*_6_ *f* complex subunit PetL	Y	N	N
3097275	3097379	+	34	smr0002	NA	Transposase, fragment	Y	N	N
146724	146831	-	35	sml0003	**psbM**	Photosystem II reaction center M protein	Y	Y	N
468997	468887	-	36	Chr_ORF_2	NA	Hypothetical protein	Y	N	N
3118192	3118302	+	36	smr0003	*petM*	Cytochrome *b*_6_ *f* complex subunit PetM	Y	N	N
**1606868**	**1606978**	**+**	**37**	**hliR1**	*hliR1*	**HliR1**	**Y**	**N**	**N**
473802	473915	-	37	sml0009	NA	VapC fragment	Y	N	Y
32865	32978	-	37	ssl5031	NA	NA	Y	N	N
831101	831217	-	38	sml0006	*rpl36*	50S ribosomal protein L36	Y	Y	Y|
1823570	1823686	+	38	smr0010	*petG*	Cytochrome *b*_6_ *f* complex subunit 5	Y	Y	N
2350140	2350256	-	38	sml0001	*psbI*	Photosystem II reaction center PsbI protein	Y	Y	N
571084	571203	+	39	smr0007	*psbL*	Photosystem II PsbL protein	Y	Y	N
571236	571355	+	39	smr0008	*psbJ*	Photosystem II PsbJ protein	Y	Y	N
1268189	1268308	-	39	sml0007	*psbY*	Photosystem II protein Y	Y	N	N
2613481	2613600	-	39	sml0002	*psbX*	Photosystem II PsbX protein	Y	N	N
2816991	2817110	-	39	sgl0001	NA	Hypothetical protein	Y	N	N
3140045	3140164	-	39	sll0047	*ycf12*	Psb30, YCF12	Y	N	N
1687326	1687448	-	40	sml0008	*psaJ*	Photosystem I subunit IX	Y	Y	N
3458023	3458145	+	40	smr0004	*psaI*	Photosystem I subunit VIII	Y	Y	N
3188105	3188227	-	40	sml0013	*ndhP*	NdhP	Y	Y	N
2138496	2138621	+	41	Chr_ORF12	NA	Hypothetical protein	Y	N	N
633626	633754	+	42	Chr_ORF5	NA	Transposase, fragment	Y	N	N
273512	273381	-	43	Chr_ORF1	NA	Hypothetical protein	Y	N	N
1167333	1167464	+	43	smr0009	*psbN*	Photosystem II PsbN protein	Y	Y	N
570940	571074	+	44	smr0006	*psbF*	Cytochrome b559 b subunit	Y	Y	N
3067172	3067306	-	44	Norf8	NA	NA	Y	N	N
30142	30008	-	44	pSYSA_ORF3	NA	N terminus of bifunctional aconitate hydratase 2/2-methylisocitrate dehydratase; similar short homologs also in other bacteria	Y	N	Y
553065	553202	-	45	sml0005	*psbK*	Photosystem II PsbK protein	Y	Y	N
1826764	1826901	+	45	smr0011	*rpl34*	50S ribosomal protein L34	Y	Y	Y
903627	903764	-	45	sml0012	NA	Hypothetical protein	Y	N	N
14477	14340	-	45	pSYSM_ORF1	NA	O-acetyl-N-acetylneuraminate esterase, partial	Y	N	N
1842716	1842856	-	46	Norf6	*ndhQ*	NdhQ subunit of the NDH-1 L complex	Y	N	N
1141803	1141946	-	47	ssl1633	*hliC, scpB*	HliC, CAB/ELIP /HLIP superfamily	Y	Y	N
**298826**	**298972**	**-**	**48**	**Norf1**	*norf1*	**Norf1**	Y	**N**	**N**
572978	573124	-	48	sml0010	NA	Transposase	Y	N	N
159812	159961	-	49	sgl0002	NA	Hypothetical protein	Y	N	N
2595112	2595264	-	50	ssl0090	NA	Hypothetical protein	Y	N	N

The start and end positions according to the chromosomal or plasmid sequences in Genbank files (accessions NC_000911, AP004311 and AP004310), the location (S) on the forward (+) or reverse strand (−) and respective length (L; in amino acids) are given, followed by the locus tag ID, gene name and product if assigned. Location on chromosome or one of the plasmids is prefixed by “Chr” or the name of the plasmid. The existence of homologs in *Synechocystis* 6714, *A. thaliana* and *E. coli* is indicated by “Y” for yes or “N” for no. Homologs tagged and detected in this study are highlighted in boldface letters. Names of genes tested in this work are in boldface

### In vivo tagging and detection of cyanobacterial μ-proteins

**Fig. 2:**
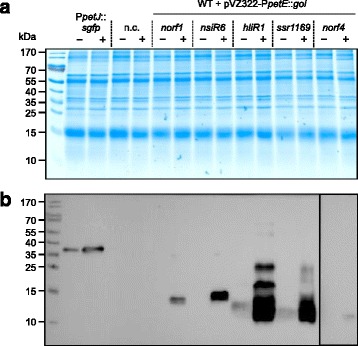
Western blot detection of small proteins. Recombinant *Synechocystis* 6803 cells carrying the genes of interest (goi) under control of the *petE* promoter on pVZ322 vector were collected before (−) or 24 h after induction of gene expression (+) for the extraction of total proteins. FLAG-tagged superfolder GFP (sfGFP) under the control of the *petJ* promoter [37] served as positive control, a WT strain carrying an empty pVZ322 vector was used as negative control (n.c.). Theoretical protein masses are listed in Table 3. Two gels were run in parallel. **a** Proteins (30 μg) were separated on a 15% (w/v) SDS polyacrylamide gel and subjected to colloidal Coomassie G-250 staining as a loading control. **b** Immunoblot with the same loading order probed with specific ANTI-FLAG® M2-Peroxidase (HRP) antibody

We chose 5 examples for closer analysis: Norf1, NsiR6, HliR1, Ssr1169 and Norf4. Norf1 and Norf4 were previously defined based on transcriptomic evidence [22]. The protein Ssr1169 was previously modelled as part of the existing annotation, but there is no information on possible functions nor that their very existence was shown thus far. NsiR6 and HliR1 are not annotated in the genome but were found by transcriptomics [21, 33]. Although these RNAs harbor potential open reading frames they were initially indicated as non-coding. After FLAG - tagging and inducing their expression in *Synechocystis* 6803, all five proteins were detected by Western blotting (Fig. 2). HliR1 and Ssr1169 showed a tendency for aggregation, even under the used denaturing conditions, possibly related to their hydrophobicity and the predicted presence of transmembrane regions (Table 3).

**Table 3:** Physicochemical properties of μ-proteins overexpressed in *Synechocystis* 6803. TMR, putative transmembrane domains, predicted using TMHMM v. 2.0 [41]

Protein	Length (aa)	MW untagged		+ FLAG tag		Predicted protein
		[kDa]	pI	[kDa]	pI	Domains
Norf4	31	3.16	5.59	5.99	4.08	1 TMR
HliR1	37	4.11	7.92	6.93	4.30	1 TMR
Norf1	48	5.47	3.76	8.30	3.88	
Ssr1169	54	6.09	7.96	8.92	4.48	Pmp3; 2 TMR
NsiR6	66	7.10	6.05	9.92	4.40	PHA02325

### The NsiR6 transcript is highly induced upon nitrogen deprivation

**Fig. 3:**
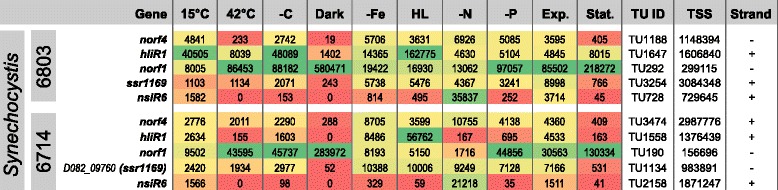
Heatmap indicating the expression of the genes encoding the five investigated small proteins in *Synechocystis* strains PCC 6803 and PCC 6714 under 10 different growth conditions: exponential (Exp.) and stationary growth phase (Stat.); cold (15 °C) and heat (42 °C) stress for 30 min each; depletion of inorganic carbon (−C), cells were washed 3 times with carbon-free BG11 and cultivated further for 20 h; dark, no light for 12 h; Fe^2+^ limitation (−Fe), addition of iron-specific chelator desferrioxamine B (DFB) and further cultivation for 24 h; high light (HL), 470 μmol photons m^−2^s^−1^ for 30 min; nitrogen depletion (−N), cells were washed 3 times with nitrogen-free BG11 and cultivated further for 12 h; phosphate depletion (−P), cells were washed 3 times with phosphate-free BG11 and further incubated for 12 h. Data derived from previous genome-wide expression analysis by differential RNA-Seq [20, 21]. Values indicate sequencing read counts for the primary 5′ end (= transcriptional start site [TSS]) of the corresponding transcriptional unit (TU). The TSS positions are given for the *Synechocystis* genomes available under accession numbers BA000022 and CP007542. The colour varies from red (no expression) to yellow (intermediate expression) to green (high expression)

**Fig. 4:**
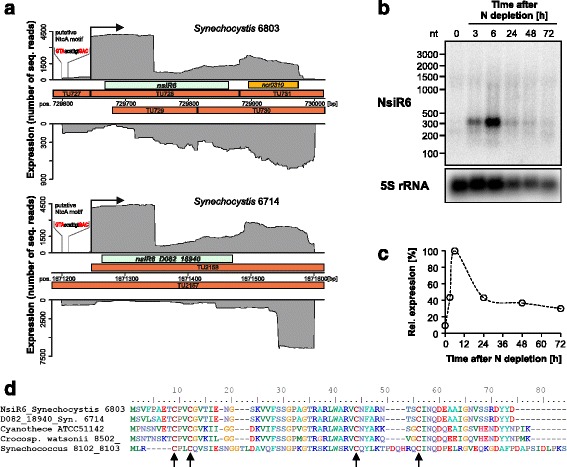
The NsiR6 peptide. **a** Transcriptomic datasets indicated high read coverage in a region without annotation in *Synechocystis* 6803 [21], which contains the here defined *nsiR6* gene. The homolog in *Synechocystis* 6714 is D082_18940 [20]. Shown is the read coverage (*grey*) resulting from previous transcriptome analysis, including the respective transcriptional units (TU) defined in that work [20, 21] and a putative NtcA binding site, centered 42 nt upstream the transcription initiation site in both strains. Relevant transcription initiation sites appear as steep increase in read coverage and are labelled by a black arrow. The length of the 5’-UTRs is 26 nt in both strains. Other non-coding TUs are colored orange. There is transcription in antisense orientation in both strains but with much lower coverage. **b** Northern blot showing the nitrogen stress-induced transcript accumulation of the NsiR6 mRNA in *Synechocystis* 6803 over 72 h. Time point 0 refers to the nitrogen-replete condition. **c** Time course of NsiR6 mRNA accumulation after normalization to 5S rRNA. The data are presented as relative to the signal at 6 h after diminishing N (=100%). **d** Sequence comparison of NsiR6 homologs from the two *Synechocystis* strains, *Cyanothece* ATCC 51142, *Crocosphaera watsonii* WH 8502 and the two marine *Synechococcus* strains WH 8102 and WH 8103 which harbor an identical protein. Four conserved cysteine residues are highlighted by arrows. These are conserved in all 63 homologs detected throughout the cyanobacterial phylum

NsiR6 was not previously known as a protein-coding gene. Its mRNA originates from a TSS at position 729645f in the chromosome of *Synechocystis* 6803 (Fig. 4a, data extracted from reference [21]). Previously, we introduced the UEF (unique expression factor) to identify genes whose expression was enhanced at a single from ten tested environmental conditions [21]. This factor gives the ratio of the transcriptome read counts for the condition with the highest and the one with the second highest expression for a single TU. Thus, TUs with a high UEF respond strongly to a particular stimulus. For NsiR6, the UEF was 9.65, ranking on position 4 of the most-strongly induced genes, both in *Synechocystis* 6803 as well as in strain 6714 [20, 21], when the cells were deprived of sources of combined nitrogen (Fig. 3). This induction was confirmed by independently performed Northern blots, indicating a rapid induction of expression, reaching a peak at 6 h with an about 10-fold higher transcript accumulation, followed by a declining abundance which remained higher than at the beginning of the experiment (Fig. 4b and c). The nitrogen-stress-dependent induction is likely mediated via a conserved NtcA binding site 5′-GTAacatttgtGAC-3′, centered 42 nt upstream the transcription initiation site in both strains (Fig. 4a). NtcA-binding sites frequently overlap the −35 promoter region and are centered close to position −41.5 with respect to the TSS when they mediate activation [23, 49]. Homologs of NsiR6 are widely conserved throughout the cyanobacterial phylum and in the *Paulinella chromatophora* chromatophore genome, consistent with its occurrence in the genomes of α-cyanobacteria, but not in any other bacteria or plants. The alignment of these homologs shows two pairs of conserved cysteine residues which might be involved in redox control, protein-protein interactions or structure formation (Fig. 4d). Two pairs of cysteine residues occur also in another short protein, the 70 amino acid CP12 protein, which mediates the formation of a complex between glyceraldehyde-3-phosphate dehydrogenase and phosphoribulokinase in response to changes in light intensity, characterizing it as a thioredoxin-mediated metabolic switch [50]. In CP12, the cysteine pairs confer the redox input via post-translational thiol-disulfide bridge conversion. The arrangement ‘CPVC’ of the first cysteine pair (Fig. 4d) matches the C-(X)_2_-C motif, which frequently is involved in metal-binding [51]. Hence, the putative cysteine pairs in NsiR6 may confer redox control or metal binding.

### Norf1 is highly induced upon dark incubation

**Fig. 5:**
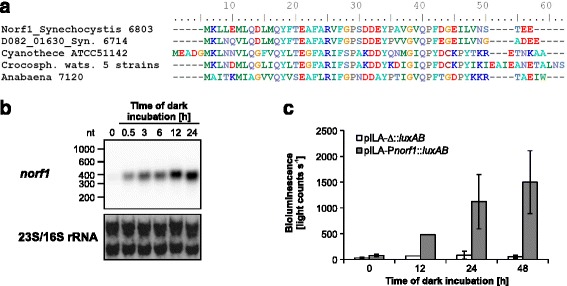
Sequence comparison and regulated expression of the *norf1* gene. **a** Sequence comparison of Norf1 homologs from the two *Synechocystis* strains, *Cyanothece* ATCC 51142, 5 different strains of *Crocosphaera* encoding an identical protein and *Anabaena* (Nostoc) sp. PCC 7120. **b** Expression of *norf1* in *Synechocystis* 6803 is strongly upregulated after transfer to darkness. **c** Bioluminescence of a *Synechocystis* 6803 reporter strain harboring a transcriptional fusion of P*norf1* (−328 to +137, TSS at +1) and *luxAB* genes in response to transfer to darkness. Representative bioluminescence dataset indicating means ± SD of measurements for two biological replicates (= independent transformants). A strain carrying a promoterless *luxAB* was used as a negative control (measured in two independent cultures each)

Norf1 is specific for cyanobacteria but widely conserved throughout this phylum. It is present in 138 (68%) of 202 cyanobacterial genomes available in the JGI database [52] (blastP + tblastN, E value ≤1e^−5^). Homologs are lacking in early-branching cyanobacteria such as *Gloeobacteria* and thermophilic *Synechococcus* JA-2-3B’a(2–13) and JA-3-3Ab and also in marine picocyanobacteria. An alignment of representative homologs is shown in Fig. 5a.

Strong accumulation of the *norf1* mRNA was observed in response to darkness (Fig. 5b). The UEF for this condition was 2.66 in *Synechocystis* 6803, but the gene was expressed also under the other tested conditions (Fig. 3) [21]. To examine whether the dark-related expression of *norf1* is under transcriptional control, we conducted reporter gene assays. The upstream sequence of *Synechocystis* 6803 *norf1* was fused to *luxAB* reporter genes encoding luciferase, and expression was monitored as bioluminescence in vivo. Indeed, the promoter activity showed a positive response after transfer into darkness as seen for the mRNA accumulation (Fig. 5b and c). We conclude that the observed induction of *norf1* in response to shifts from light exposure to darkness is under transcriptional control.

The high expression of the *norf1* gene in darkness sets it apart from the vast majority of genes. Among the previously tested 10 different growth conditions, in *Synechocystis* 6803 only 70 out of 4091 TUs and in *Synechocystis* 6714 only 57 out of 4292 TUs defined in total had their maximum expression after dark incubation [20, 21].

### The Norf4 μ-protein is highly conserved and its mRNA overlaps the *gap1* gene

**Fig. 6:**
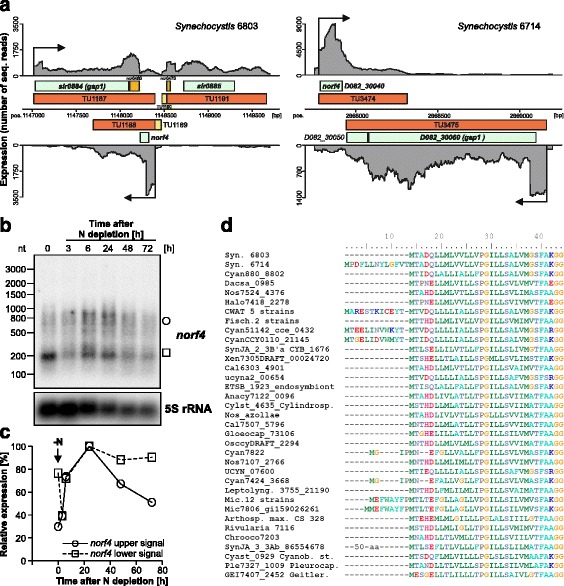
The Norf4 peptide. **a** Datasets from the previously performed primary transcriptome analysis showed that Norf4 expression responded positively to nitrogen depletion in both *Synechocystis* 6803 [21] and *Synechocystis* 6714 [20], when 11 different growth conditions were tested. The mRNAs of *norf4* and *gap1* are co-regulated and overlap by several hundred nt. The previously mapped transcriptional start sites are labelled by *black arrows*. **b** Northern blot analysis of *norf4* mRNA accumulation in a time course experiment up to 72 h after the removal of nitrogen. The same RNA samples were used as in Fig. 4. **c** The signals obtained from the Northern blots (panel **b**) were evaluated densitometrically after normalization by the level of 5S rRNA. The relative *norf4* expression is shown with respect to the maximum expression after transfer to nitrogen-free conditions (24 h = 100%). The bands at 200 nt (filled circles) and at 800 nt (empty circles) were analyzed separately from each other. **d** Multiple sequence alignment of 35 homologs from 51 different cyanobacterial genome sequences (homologs are identical among 12 *Microcystis*, five *Crocosphaera watsonii* and two *Fischerella* genome sequences)

Norf4 is encoded within a TU much longer than is needed to encode the 31 amino acids: TU1188 in *Synechocystis* 6803 is 704 nt and TU3474 in *Synechocystis* 6714 is 534 nt (Fig. 6a). These TUs partially overlap the *gap1* gene encoding glyceraldehyde 3-phosphate dehydrogenase 1 on the complementary DNA strand. As a result, these TUs overlap the *gap1* mRNA by 702 and 373 nt, respectively. Transcriptomic evidence suggested that both the *gap1* and the *norf4* mRNAs were co-regulated with each other, with a mild up-regulation upon the removal of nitrogen (Fig. 3). Thus, the *norf4* transcript does not function as an antisense RNA with a co-degradation function, which was observed previously for other pairs of overlapping transcripts in *Synechocystis* 6803 [53, 54]. However, co-regulation between an asRNA and its cognate mRNA was previously observed for the *psbA* asRNA protecting its 5′ leader from RNase E-mediated degradation [55]. The expression of *norf4* was stimulated upon removal of nitrogen, but its expression was detectable under most of the previously tested conditions, although at a lower level (especially low in darkness and after heat stress; Fig. 3). Dual-function RNAs are transcripts which assume a regulatory function as sRNA and additionally act as short protein-coding mRNA. Exploring this possibility for *norf4*, we checked the accumulation of *norf4* transcripts during the removal of combined nitrogen. Northern blot analysis showed the existence of a prominent transcript of ~200 nt which declined initially (Fig. 6b). Due to the localization of the RNA probe used in the detection of *norf4* transcripts, this prominent transcript corresponds to the coding part of TU1188. However, with increasing duration of the nitrogen stress, we noticed the overaccumulation of a longer transcript, of about 600–800 nt that appeared more diffuse (Fig. 6b). Quantitative analysis of transcript accumulation showed that this longer *norf4* transcript was only transiently accumulated, with a peak at the 24 h time points (Fig. 6c).

With very few amino acid substitutions, Norf4 is extremely conserved, including a predicted transmembrane region (Fig. 6d). Homologs can be detected in 51 cyanobacterial genome sequences from all 5 morphological subsections, comprising free-living unicellular as well as multicellular strains, marine and freshwater isolates, thermophiles and symbionts. The presence of *norf4* in the two available genome sequences of *Candidatus* Atelocyanobacterium thalassa suggests their positive selection in these highly streamlined genomes [56, 57]. However, homologs are lacking in α-cyanobacteria, which are mainly marine *Synechococcus* and *Prochlorococcus*. The homologs from the two used *Synechocystis* strains are identical, except for a possible N-terminal extension by 13 amino acids in *Synechocystis* 6714 (Fig. 6d). However, such extensions appear questionable also in other strains, because the start codon corresponding to the *Synechocystis* 6803 ORF is 100% conserved. Moreover, the homologs in 12 *Microcystis* genomes are identical to each other, as are the homologs in five *Crocosphaera watsonii* and in two *Fischerella* genome sequences.

Our data suggest that Norf4 is a previously unknown membrane-bound μ-protein and that the *norf4* transcript may play a dual role, with a mainly coding function during nitrogen-sufficient conditions and a possibly RNA-mediated regulatory function on the *gap1* mRNA during nitrogen stress.

### HliR1 and Ssr1169

**Fig. 7:**
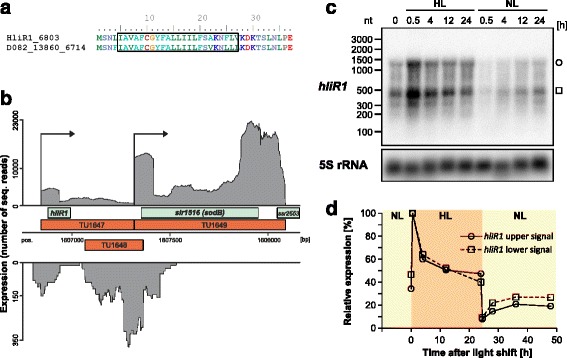
The HliR1 peptide in *Synechocystis* 6803. **a** Pairwise sequence alignment of the HliR1 peptides from *Synechocystis* 6803 and *Synechocystis* 6714. A predicted transmembrane region is boxed. **b** Data replotted from the primary transcriptome analysis of *Synechocystis* 6803 suggest that HliR1 expression is induced by high light and that transcripts may extend into the subsequent TU1649 covering the *sodB* gene [21]. **c** Northern analysis of *hliR1* mRNA accumulation upon transfer to high light (HL) or normal light (NL). **d** Quantification of the *hliR1* mRNA accumulation shown in panel **c** after normalization to the 5S rRNA level. Relative values refer to the maximum level at 0.5 h after HL shift (=100%)

HliR1 was chosen because of its very high induction under high light (UEF of 5.47) and the gene location upstream of *sodB* encoding superoxide dismutase. Whereas the homologs from the two *Synechocystis* strains are conserved in length, sequence (2 substitutions over 35 amino acids) and the likely presence of a transmembrane region (Fig. 7a), no possible homologs were detected beyond the genus *Synechocystis*. The location upstream of *sodB* and the shape of the read coverage in transcriptome analysis (Fig. 7b) suggested a possible link between the two genes. Indeed, Northern analysis confirmed the inducibility by high light (Fig. 7c and d) and in addition showed the presence of two major transcripts, ~450 and 1400 nt in length. The longer form should encompass also the complete *sodB* gene. Thus, transcription from the upstream located *hliR1* promoter will lead by read-though to an enhanced *sodB* gene expression under high light. Hence, it is tempting to speculate, that HliR1 is a membrane-bound peptide with a regulatory function on the superoxide dismutase.

The previously annotated short gene *ssr1169* was chosen because of its expression under several different conditions (Fig. 3) and its physicochemical characterization as a hydrophobic protein. Features of all 5 investigated μ-proteins are summarized in Table 3.

**Fig. 8:**
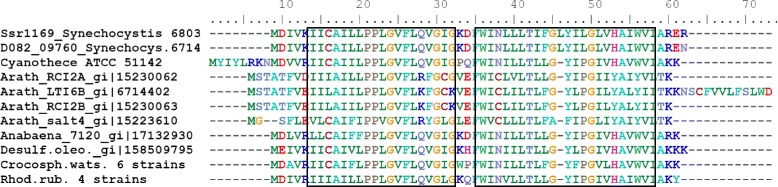
Sequence alignment of Ssr1169 homologs from cyanobacteria with those from *Arabidopsis thaliana*, *Desulfococcus oleovorans* Hxd3, and identical proteins in 4 strains of *Rhodospirillum rubrum*. Putative transmembrane domains were predicted using TMHMM v. 2.0 [41] and are *boxed*

Homologs of Ssr1169 are frequently encoded by a small gene family and exist in plants (best homolog in *A. thaliana*: Low temperature and salt responsive protein, gi|15223610|ref|NP_176067.1|, E value 3e^-11;^ Table 2; Fig. 8), in *E. coli* (gi|446430313|ref|WP_000508168.1|, E value 3e^−8^; Table 2) and in many other bacteria and other eukaryotic organisms, including yeast and *C. elegans*. Expression of the homologs RCI2A and RCI2B in *A. thaliana* became induced upon exposure to low temperature, dehydration, salt stress, or abscisic acid [58]. Ssr1169 homologs possess two transmembrane helices (Fig. 8) that form a Pmp3 domain and might be a stress induced proteolipid membrane modulator.

### All five μ-proteins can be expressed from their native promoters in a regulated fashion

**Fig. 9:**
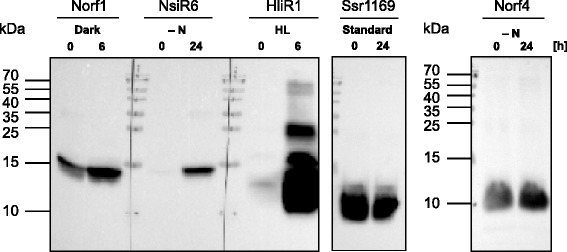
Detection of μ-proteins upon expression from their native promoters and 5′ UTRs. Recombinant *Synechocystis* 6803 cells carrying the respective genes under control of their own promoters and 5′UTRs on vector pVZ322 were collected from cultures grown at standard conditions (0) and after transfer to the respective inducing condition at indicated time points (6 or 24 h) or in case of Ssr1169 after 24 h at standard condition. The Western blot was probed with specific ANTI-FLAG® M2-Peroxidase (HRP) antibody. All samples were separated on the same gel and transferred to the same membrane but the part probed for Norf4 had to be exposed longer because of its lower expression. Prestained Protein Ladder (10–170 kDa, Fermentas) was used as molecular weight marker

In the previous sections we verified the transcription of the five selected μ-protein encoding genes (Figs. 3, 4, 5, 6 and 7) as well as their translation from an mRNA harboring the regulatory sequence elements (e.g. ribosome binding site) of the *petE* gene (Fig. 2). However, despite verifying a stable accumulation of the translated protein the latter approach renders the possibility of translating all RNAs as long as they contain an open reading frame. To exclude this possibility, we repeated the experiment from Fig. 2 but placed all five FLAG-tagged μ-ORFs under control of their own, native promoter and 5′UTRs. After introduction of these constructs into *Synechocystis* 6803 we subjected the resulting cultures to an inducing condition according to the transcriptome analysis. Samples from cultures grown at standard conditions or the inducing conditions were taken and analyzed by Western blot experiments (Fig. 9). The results showed unambiguously the expression of all five μ-proteins when placed under control of their own promoters and 5′ UTRs, i.e., their expression was not artificially induced by the ectopic fusion of their ORFs to the *petE* promoter and 5′ UTR. We noticed a strong upregulation of NsiR6 accumulation 24 h after transfer to nitrogen starvation and of HliR1 accumulation 6 h after exposure to high light as well as a mild upregulation of Norf4 accumulation 24 h after transfer to nitrogen starvation (Fig. 9). The accumulation of Norf1 increased somewhat 6 h after the shift to darkness. These data show that the observed regulation of gene expression at RNA level has a strong effect on the amounts of three of the respective proteins and a milder on one of the other two.

## Discussion

For *Synechocystis* 6803 alone, more than 50 independent proteomic studies identified a total of 2967 proteins at least once (reviewed by Gao et al., [59]), representing 80.8% of the entire predicted proteome. However, the percentage of identified proteins was only 34.4% for small proteins (<100 aa) of high hydrophobicity [59]. In addition, as we show in this study, very short protein-coding genes might not even be modelled and annotated at all. Thus, due to the challenges in their identification and biochemical detection, μ-proteins were in the past either not detected or were ignored. However, systematic genome-wide approaches have recently reported an increasing number of μ-proteins in pro- and eukaryotes [8, 10, 11, 19, 60]. Besides the short ORFs within 5′ leader and 3′ trailer sequences of mRNAs, known for a long time [61–65], μ-peptides were recently also described to originate from long ncRNAs, i.e. transcripts, which were previously assumed to be non-coding [60, 66].

In *E. coli* approximately 60 genes encoding μ-proteins have previously been reported [67]. Expression profiling showed that many μ-proteins accumulate under specific growth conditions or are induced by stress [68]. A particular group of small proteins are toxic due to their integration into the cell membrane as peptide component of a type I toxin-antitoxin system [69–71]. In the cyanobacterium *Synechococcus elongatus*, four small secreted proteins have been suggested to be involved in biofilm development [72]. Small proteins of the type II toxin-antitoxin category in *Synechocystis* 6803 have been catalogued separately [73] but the majority of them are somewhat larger than the here considered μ-proteins.

Here, we found 293 candidate genes for small proteins ≤80 amino acids in the model cyanobacterium *Synechocystis* 6803 and demonstrate the synthesis of five examples by C-terminal FLAG-tagging and immune detection. Three of these five small proteins are predicted to contain one or two transmembrane helices (Table 3), placing them in the category of proteins that are particularly challenging to verify by proteomic approaches [59]. Hence, our list of predicted proteins provides a solid basis for functional studies.

Regulated expression suggests involvement in stress adaptation for some of the here investigated small proteins. This applies especially to HliR1, NsiR6 and Norf1, whose expression is activated in response to high light, nitrogen stress or transfer into darkness (Figs. 3, 4, 5 and 9).

The fact that some of the here described proteins are part of TUs much longer than needed points to the possibility that some of them could constitute dual function RNAs. Such dual-function RNAs that in addition to their role as a regulatory RNA molecule also encode a functional peptide, have been identified in bacteria. A prominent example for a dual function RNA is the 43 amino acid peptide SgrT encoded in the 5′ region of the *E. coli* SgrS transcript, which regulates the glucose transporter PtsG at protein level, whilst the SgrS 3′ region contains a regulatory domain that targets the *ptsG* mRNA by base-pairing [74].

In *Bacillus subtilis*, SR1 is a highly conserved dual-function sRNA that acts as a base-pairing regulatory RNA on the *ahrC* mRNA (encoding AhrC, the transcriptional activator of arginine catabolic operons) and in addition encodes the 39 amino acid peptide SR1P. Interestingly, this peptide binds GapA (glyceraldehyde-3-phosphate dehydrogenase), thereby stabilizing the *gapA* operon mRNA [75, 76]. In analogy, it is interesting to note that the here described cyanobacterial Norf4 μ-protein overlaps the *gap1* mRNA and appears to be co-regulated with it.

The high total numbers of predicted μ-ORFs, together with the distribution, conservation, regulation of gene expression and the physicochemical properties of the five examples studied here in more detail, underline the likely great bandwidth of small protein functions in bacteria and makes them attractive candidates for functional studies.

## Conclusions

*Synechocystis* 6803 is a widely used model cyanobacterium that possess with 44 genes encoding small proteins ≤50 amino acids and potentially 293 proteins ≤80 amino acids a high number of such μ-ORFs. These numbers are certainly no overestimation: due to the previous extensive work to elucidate all subunits of the photosynthetic apparatus, 52% of the small proteins ≤50 amino acids have a known function. This sets the small proteome of cyanobacteria apart from that of other bacteria: in addition to the 19 photosynthesis-related small proteins only five other in the size category ≤50 are functionally annotated (NdhP,NdhQ, RpL34, Rpl36 and a VapC toxin homolog). Hence, about half of the predicted small proteins are uncharacterized. When analysing small proteins up to 80 aa, we found 235 of the 293 predicted small proteins (80%) without annotation. The experimental results and expression data for the five here selected proteins (three ≤50 aa and another two larger, but ≤70 aa) underline that it is worthwhile to study small protein functions directly in cyanobacteria. The here provided data and strains will be useful for such studies in a systematic way.

## Abbreviations

HliR1: High light inducible RNA 1
Norf1 and Norf4: Novel orf 1 and 4
NsiR6: Nitrogen stress-induced RNA 6
TU: Transcriptional unit
UEF: Unique expression factor
